# Short-term insomnia symptom profile transitions in older patients with cancer pain

**DOI:** 10.1016/j.isci.2026.116557

**Published:** 2026-07-01

**Authors:** Hongyu Zhu, Rong Qi, Huiyu Luo, Huiqun Chen, Meiqun Lin, Xi Ke, Wenting Huang, Liqin Jiang, Yunzhen Peng

**Affiliations:** 1 Clinical Oncology School of Fujian Medical University &Fujian Cancer Hospital, Fuzhou, Fujian, China; 2 The School of Nursing, Fujian Medical University, Fuzhou, Fujian, China; 3 Fujian Agriculture and Forestry University, Fuzhou, Fujian, China

**Keywords:** aging, oncology, sleep medicine, clinical epidemiology

## Abstract

Insomnia symptoms are common in older adults with cancer pain and may fluctuate during hospitalization. This longitudinal study explored short-term changes in insomnia symptom profiles in 238 patients aged 60 years or older across two assessments conducted within 48 h of hospital admission and again 7–10 days after treatment initiation. Using the Athens Insomnia Scale, we identified four profiles: minimal or no insomnia symptoms, daytime dysfunction, low sleep efficiency, and high insomnia symptoms. Profile membership showed short-term reclassification, with the High Insomnia Symptoms profile remaining relatively stable and some patients with milder or domain-specific symptoms moving to more severe profiles. Higher baseline pain, fatigue, and anxiety were associated with an increased likelihood of short-term worsening in insomnia symptom profiles, whereas lower pain burden and better physical performance were associated with more favorable insomnia profiles. These findings highlight heterogeneous, short-term fluctuations in insomnia symptoms among older patients with cancer pain.

## Highlights

• Four distinct insomnia profiles were identified in older cancer pain patients
• Insomnia symptom profiles showed short-term fluctuation over follow-up
• Baseline pain, fatigue, and anxiety predicted worsening insomnia symptoms
• Better physical function was associated with more favorable insomnia profiles

## Graphical abstract

**Figure:**
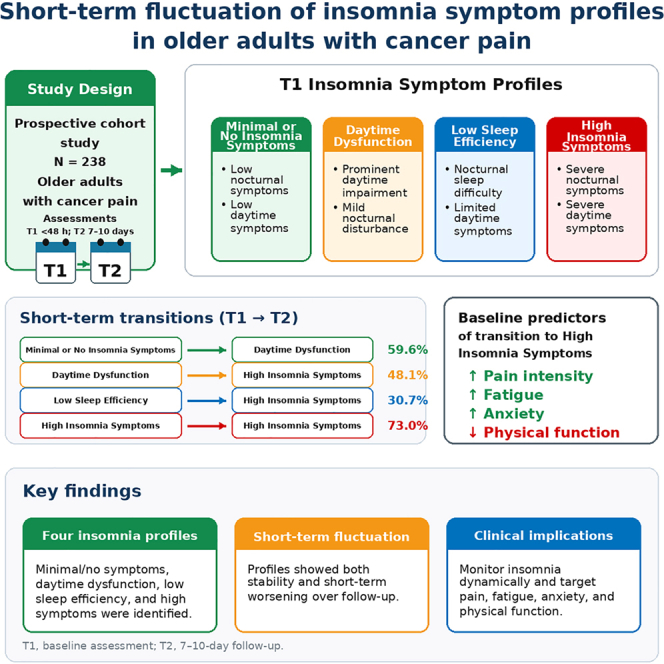

## Teaser

Health sciences.

## Introduction

According to data from the US National Statistical Office, the number of people aged 65 and older is expected to double in the next 25 years.^1^ With the rapid aging of the global population, the number of older cancer patients is steadily increasing. Cancer-related pain remains one of the most common and distressing symptoms in this population, affecting about 30%–50% of cancer patients at various stages of the disease.^2^ Persistent pain in older adults not only impairs their physical health but also has a profound impact on their mental health and overall quality of life.^3^ In addition to pain, insomnia is highly prevalent among cancer patients. Insomnia is a sleep disorder characterized by difficulty falling asleep or maintaining sleep, which can lead to distress and negatively affect daily life. It affects about 6%–10% of the general population, with a prevalence three times higher among cancer patients and cancer survivors.^4^ A cohort study in China showed that about 66.7% of patients with pain had insomnia, compared with 33.3% of those without pain. Patients who had both insomnia and pain also reported more severe symptoms. These symptoms included fatigue, emotional distress, and nerve pain. Together, these findings suggest that insomnia is both more common and more severe in cancer patients with pain.^5^ Due to age-related physiological frailty and multiple comorbidities, the risk is especially high for older patients. Beyond its impact on quality of life, insomnia may also affect the course of co-existing symptoms and clinical outcomes. Poor sleep has been associated with worse pain control, increased fatigue, and reduced adherence to cancer treatment, which may ultimately compromise overall treatment effectiveness and survival.^6^

Despite its significant clinical implications, the natural course of symptoms for insomnia in elderly cancer patients remains unclear. Previous studies have shown that sleep issues in cancer patients are dynamic: some patients experience persistent insomnia, while others may improve or worsen over time.^7^ However, traditional research methods that focus on averages often overlook individual differences. This limitation highlights the need to use a person-centered analysis to identify subgroups with distinct symptom patterns. In this context, latent profile analysis (LPA) is a statistical method that helps identify hidden patterns based on a set of indicators.^8^ Typically, research designs that focus on variables explain how these variables relate to each other and assume that these relationships apply to all individuals. In contrast, LPA focuses on grouping individuals based on their symptom profiles rather than analyzing variable correlations. LPA allows us to group people into profiles based on probabilities, with individuals in each profile sharing similar traits, thereby helping us identify potential subgroups.^9^

To explain the multiple aspects and natural heterogeneity of insomnia symptoms, a person-centered longitudinal analysis approach is required. Importantly, LPA can be extended to model longitudinal data through latent profile transition analysis (LPTA). LPTA is a flexible analytical tool suitable for both cross-sectional and longitudinal person-centered research; it can estimate group membership and changes in subgroup membership or characteristics over time.^10^ In LPTA, the focus is on assessing the transitions among latent profile members over time, which allows us to clarify the stability or variability of insomnia profiles among subtypes.^8^^,^^11^

Identifying factors associated with insomnia symptom profiles is crucial for recognizing individuals at higher risk. Existing studies have shown that insomnia is closely related to various clinical and demographic factors. Previous research indicates that sex differences are prominent in insomnia, with women more likely than men to experience it; this may be linked to hormonal and psychosocial factors.^12^^,^^13^ Living arrangements are also linked to sleep quality. Chu et al. study suggests that elderly women living alone are more likely to feel lonely and lack support, resulting in a higher risk of poor sleep quality compared to those who live with others.^13^ Pain is a core predictive factor for insomnia in cancer patients, as increased pain intensity significantly worsens difficulties in falling asleep and increases nighttime awakenings; this may be related to the stress and psychological distress caused by pain. Additionally, opioids can disrupt circadian rhythm regulation. Long-term and high-dose opioid use may worsen sleep quality by altering sleep structure and circadian rhythms.^14^ Furthermore, cancer-related fatigue (CRF) has a bidirectional relationship with insomnia: higher levels of fatigue are often found alongside more severe insomnia, and fatigue frequently exacerbates difficulties in falling asleep and the frequency of nighttime awakenings, thereby triggering or maintaining insomnia.^15^^,^^16^ Pain can also impact sleep quality, contributing to CRF.^17^ Anxiety is another significant psychological predictor; anxiety symptoms can induce insomnia by increasing arousal levels and emotional distress.^18^ Several biological mechanisms explain the complex bidirectional interactions among cancer-related insomnia, anxiety, and fatigue, including cytokines and hypothalamic-pituitary-adrenal (HPA) axis dysregulation.^19^ Chong et al. study indicates that better physical functioning reduces the likelihood of insomnia by promoting daytime activity levels and regulating circadian rhythms.^20^ To determine whether these specific individual factors are related to the profiles and patterns of insomnia symptoms, we report an analysis of the following baseline variables linked to insomnia profiles and transition probabilities: sex, living arrangements, pain intensity, weekly cumulative morphine-equivalent dose, fatigue, and anxiety. This analysis helps clarify how symptoms interact in cancer pain patients and provides a theoretical basis for individualized interventions in clinical practice.

Our research has two main goals: (1) to identify distinct insomnia symptom profiles among older patients with cancer pain and (2) to examine short-term fluctuation in profile membership across two assessments and the baseline factors associated with these fluctuations.

## Methods

This study employed a prospective cohort design to investigate short-term fluctuations in insomnia symptom profiles among older adults with cancer pain. Detailed methodological information is provided in the STAR Methods section.

## Results

### Assessment of common method bias

Common method bias was assessed using Harman’s one-factor test.^21^ An unrotated principal-component analysis was conducted for data at both time points (T1 and T2). The results extracted two factors with eigenvalues greater than 1 at each time point. The first factor accounted for 29.92% of variance at T1 and 30.14% at T2, both below the critical threshold of 40%. These findings suggest that common method bias was unlikely to have affected the results.

### Participants’ characteristics and symptom severity scores

Of the 238 respondents, the mean age was 67.94 years (standard deviation [SD] = 6.12; range 60–95). 11 patients (4.6%) were in stage II, 83 patients (34.9%) were in stage III, and 144 patients (60.5%) were in stage IV. The distribution of tumor types was as follows: 89 participants (37.4%) had digestive system tumors, 92 participants (38.7%) had respiratory system tumors, 25 participants (10.5%) had gynecological or breast tumors, and 32 participants (13.4%) had other types of tumors. Regarding education, 75.5% of participants had an education level of junior high school or below, 20.4% had completed high school or vocational education, and 4.1% had a bachelor’s degree or higher. 144 (60.5%) were male. Most participants (59.2%) lived with family. 55 patients (23.1%) received a weekly cumulative morphine dose of 0–100 mg. Variance inflation factor (VIF) diagnostics showed that all VIF values among pain, fatigue, and anxiety were below 1.5, indicating no significant multicollinearity issues. The mean Numeric Rating Scale (NRS) pain score was 4.53 (SD = 1.31), with 28.6% reporting mild, 64.3% moderate, and 7.1% severe pain. The mean Revised Piper Fatigue Scale (RPFS) score was 5.64 (SD = 2.23); 20.2% reported mild fatigue, 47.0% moderate, and 32.8% severe fatigue. The mean Generalized Anxiety Disorder 7-item scale (GAD-7) score was 12.79 (SD = 4.76), with 96.2% showing potential anxiety (21.0% mild, 39.5% moderate, and 35.7% severe). The mean Short Physical Performance Battery (SPPB) score was 7.05 (SD = 3.15), with 41.2% classified as having poor, 36.1% moderate, and 22.7% good physical performance (see Table 1).

**Table 1:** Descriptive statistics of all covariates used in LPTA (*n* = 238)

Covariates	Code	Label	*n* (%)	Mean, SD (range)
Sex	1	male	144 (60.5)	–
	2	female	94 (39.5)	–
Living arrangements	0	living alone or in a care facility	97 (40.8)	–
	1	living with family	141 (59.2)	–
Average pain intensity	–	–	–	4.53, 1.31 (2–8)
Weekly cumulative morphine-equivalent dose	0	0–100 mg	55 (23.1)	–
	1	101–500 mg	106 (44.5)	–
	2	>500 mg	77 (32.4)	–
RPFS	–	–	–	5.64, 2.23 (0.6–10)
GAD-7	–	–	–	12.79, 4.76 (0–21)
SPPB	–	–	–	7.05, 3.15 (0–12)

SD, standard deviation.

Table 2 presents the prevalence of positive responses on individual Athens Insomnia Scale (AIS) items and total AIS score at T1 and T2. At T1, 95.7% (228/238) of older patients with cancer pain had AIS scores >6, indicating clinically relevant insomnia symptoms. At T2, this proportion increased to 97.4% (232/238), demonstrating a short-term increase in insomnia prevalence over the follow-up period. However, the difference in overall prevalence between the two time points was not statistically significant (X^2^ = 1.035, *p* = 0.309), suggesting no significant change in the population-level rate of insomnia. Nevertheless, this does not preclude potential short-term fluctuation in insomnia symptoms at the individual level, which warrants further exploration through latent transition analysis.

**Table 2:** Presents the prevalence of positive responses on individual AIS items and total AIS score at T1 and T2 (*n* = 238)

AIS Item	T1	T2	X^2^	*p value*
Item 1	89.50%	93.28%	2.162	0.141
Item 2	89.92%	92.44%	0.940	0.332
Item 3	89.08%	90.34%	0.205	0.651
Item 4	89.50%	92.44%	1.253	0.263
Item 5	85.29%	91.60%	4.625	0.032
Item 6	85.29%	91.18%	3.967	0.046
Item 7	84.03%	87.82%	1.407	0.236
Item 8	87.39%	90.34%	1.040	0.308
AIS total score >6	95.7%	97.4%	1.035	0.309

The items listed in this table correspond to specific domains of AIS. The AIS is designed to assess insomnia severity as a whole, and individual item scores should be considered in the context of the total AIS score. AIS items: Item 1: sleep induction; Item 2: awakenings during the night; Item 3: final awakening; Item 4: total sleep duration; Item 5: sleep quality; Item 6: well-being during the day; Item 7: functioning capacity during the day; Item 8: sleepiness during the day.

### Cross-sectional LPA of insomnia symptom fluctuation in older adults with cancer pain

First, cross-sectional LPA was performed at two waves. Fit indices indicated that the four-class model provided the lowest Information Criterion（IC） values and relatively high entropy. According to Lubke,^22^ entropy values should reach 0.80 and above, which means at least 90% correct assignment. On the other hand, heterogeneity between classes decreases as more classes are added.^23^ It is important to consider not only the consistency across the two waves but also the parsimony of the model. To achieve a balance between theoretical relevance and statistical considerations, the four-class model was chosen (see Table 3).

**Table 3:** Fit indices for LPA models with 1–5 classes in the two waves

Time	No. of classes	AIC	BIC	aBIC	Entropy	LMR	BLRT
Time 1	1	5,603.147	5,658.703	5,607.989	–	–	–
	2	5,361.326	5,448.133	5,368.891	0.835	<0.001	<0.001
	3	5,299.342	5,417.399	5,309.630	0.880	<0.001	<0.001
	4	5,259.334	5,408.642	5,272.345	0.877	<0.001	<0.001
	5	5,246.365	5,426.923	5,262.100	0.900	0.220	0.215
Time 2	1	5,286.047	5,341.603	5,290.888	–	–	–
	2	5,026.316	5,113.123	5,033.880	0.901	0.0133	0.0123
	3	4,968.171	5,086.228	4,978.459	0.776	<0.001	0.0173
	4	4,904.890	5,054.197	4,917.901	0.904	0.0188	<0.001
	5	4,768.325	5,073.885	4,794.953	0.903	0.3910	0.3828

**Figure 1:**
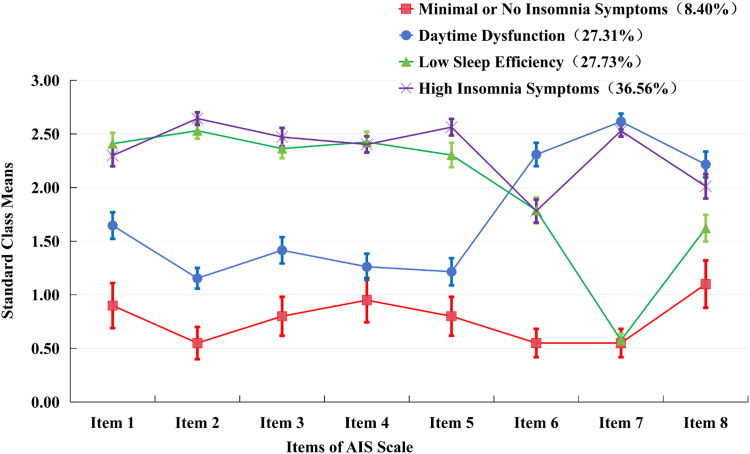
Standard class means for four-class solution in T1 Points indicate class-specific mean scores for each Athens Insomnia Scale item, and error bars indicate standard errors (SEs). Item 1: sleep induction; Item 2: awakenings during the night; Item 3: final awakening; Item 4: total sleep duration; Item 5: sleep quality; Item 6: well-being during the day; Item 7: functioning capacity during the day; Item 8: sleepiness during the day. *n* = 238.

**Figure 2:**
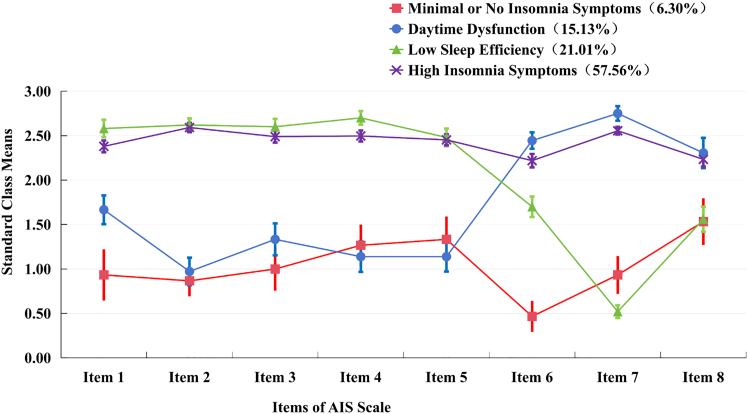
Standard class means for four-class solution in T2 Points indicate class-specific mean scores for each Athens Insomnia Scale item, and error bars indicate standard errors (SEs). Item 1: sleep induction; Item 2: awakenings during the night; Item 3: final awakening; Item 4: total sleep duration; Item 5: sleep quality; Item 6: well-being during the day; Item 7: functioning capacity during the day; Item 8: sleepiness during the day. *n* = 238.

The insomnia symptom fluctuation profiles were similar across the two waves (see Figures 1 and 2). The four profiles were labeled as minimal or no insomnia symptoms, daytime dysfunction, low sleep efficiency, and high insomnia symptoms. The results were based on cross-sectional analyses. Still, they help describe patterns of change in insomnia symptom fluctuation from an inter-individual perspective.

In both waves, the Minimal or No Insomnia Symptoms profile included 20 participants at T1 (8.40%) and 15 participants at T2 (6.30%). Individuals in this group reported low scores on all AIS items. At T1, 10 participants scored ≤6 on the AIS, which means they had little to no insomnia. The other 10 participants scored >6, which showed they had mild insomnia symptoms. At T2, 6 participants had scores ≤6, while 9 participants scored >6. The Daytime Dysfunction profile included 65 participants at T1 (27.31%) and 36 participants at T2 (15.13%). This group showed high scores on daytime-related items. Scores were elevated for well-being during the day (T1: mean 2.31, SD = 0.88; T2: mean 2.44, SD = 0.55), functioning capacity during the day (T1: mean 2.62, SD = 0.60; T2: mean 2.75, SD = 0.49), and sleepiness during the day (T1: mean 2.22, SD = 0.97; T2: mean 2.31, SD = 1.02). Scores on the other AIS items were lower. The Low Sleep Efficiency profile included 66 participants at T1 (27.73%) and 50 participants at T2 (21.01%). This group showed high scores on nighttime sleep items. Scores were elevated for sleep induction (T1: mean 2.41, SD = 0.82; T2: mean 2.58, SD = 0.70), awakenings during the night (T1: mean 2.53, SD = 0.61; T2: mean 2.62, SD = 0.52), final awakening (T1: mean 2.36, SD = 0.73; T2: mean 2.60, SD = 0.63), total sleep duration (T1: mean 2.42, SD = 0.78; T2: mean 2.70, SD = 0.54), and sleep quality (T1: mean 2.30, SD = 0.92; T2: mean 2.48, SD = 0.70). Scores on the remaining items were lower. The High Insomnia Symptoms profile included 87 participants at T1 (36.56%) and 137 participants at T2 (57.56%). Participants in this group reported high scores on all AIS items.

### LPTAs of insomnia symptom fluctuation in older adults with cancer pain

#### Latent profile transitions across time

The transition probabilities (see Table 4) reflected the probability of exhibiting a particular insomnia symptom fluctuation profile at time t + 1 conditional on the insomnia symptom fluctuation profile at time t. Thus, the diagonal elements represented the proportion of older adults with cancer pain who were classified in the same insomnia symptom profile at both assessments. For example, individuals classified in the High Insomnia Symptoms profile at time 1 had a high probability of being classified in the same profile at time 2 (transition probability = 0.730), suggesting limited short-term fluctuation in insomnia symptoms in this group and minimal symptom improvement. The transition probabilities for the Minimal or No Insomnia Symptoms, Daytime Dysfunction, and Low Sleep Efficiency latent statuses were 0.223, 0.300, and 0.180, respectively, indicating that some individuals experienced changes in their insomnia symptom fluctuation profiles. Notably, individuals in the Minimal or No Insomnia Symptoms latent status at T1 had a probability of transitioning to the Daytime Dysfunction status at T2 (transition probability = 0.596). Similarly, individuals classified as Daytime Dysfunction and Low Sleep Efficiency at T1 were more likely to fluctuate to the High Insomnia Symptoms status at T2, with transition probabilities of 0.481 and 0.307, respectively. The overall results suggested that insomnia symptom fluctuation profiles showed short-term variation between assessments.

**Table 4:** Transition probabilities for the latent profile transition analysis model

Time	Minimal or No Insomnia Symptoms	Daytime Dysfunction	Low Sleep Efficiency	High Insomnia Symptoms
Time 1	0.08	0.27	0.28	0.37
Time 2	0.06	0.15	0.21	0.58
Minimal or No Insomnia Symptoms	**0.223**	0.596	0.097	0.084
Daytime Dysfunction	0.158	**0.300**	0.061	0.481
Low Sleep Efficiency	0.060	0.030	**0.180**	0.307
High Insomnia Symptoms	0.196	0.176	0.321	**0.730**

Transition probabilities in bold font correspond to membership in the same latent status at each wave; latent statuses at T1 are presented in rows, and latent statuses at T2 are presented in columns.

#### Incorporating covariates

Seven covariates (sex, living arrangements, average pain intensity, weekly cumulative morphine-equivalent dose, fatigue, anxiety, and physical performance) were added into this LPTA model to explore whether individual characteristics affected transitions in insomnia symptom fluctuation over time.

Using the Minimal or No Insomnia Symptoms at T1 as the reference category, odds ratios (ORs) for transitions into the Daytime Dysfunction, Low Sleep Efficiency, and High Insomnia Symptoms were estimated. The results indicated that sex, living arrangement, and weekly cumulative morphine-equivalent dose had no significant effects on changes in insomnia symptom fluctuation (*p* > 0.05). In contrast, average pain intensity, fatigue, anxiety, and physical performance significantly influenced transitions in insomnia symptom fluctuation (*p* < 0.05). Except for physical performance, all other covariates had ORs greater than 1, suggesting that greater intensity of pain (OR = 2.27, 1.99, 3.09), fatigue (OR = 1.59, 2.27, 2.04), and anxiety (OR = 1.14, 1.21, 1.16) was associated with a greater likelihood of being classified in more severe insomnia symptom profiles at follow-up. Conversely, physical performance had ORs less than 1 (OR = 0.55, 0.60), indicating that better physical performance was associated with a reduced risk of worsening insomnia symptom fluctuation (see Table 5).

**Table 5:** Odds ratios of T1 latent class membership as a function of covariates

Covariates	Minimal or No Insomnia Symptoms	Daytime Dysfunction	Low Sleep Efficiency	High Insomnia Symptoms	*p* value
Sex	REF	1.33	2.09	1.77	0.723
Living arrangements	REF	0.91	1.42	0.44	0.287
Average pain intensity	REF	2.27^a^	1.99^a^	3.09^b^	<0.001
Weekly cumulative morphine-equivalent dose	REF	1.51	2.21	1.67	0.083
RPFS	REF	1.59^a^	2.27^b^	2.04^b^	<0.001
GAD-7	REF	1.14^b^	1.21^a^	1.16^a^	<0.001
SPPB	REF	0.55^b^	0.873	0.60^a^	<0.001

a *p* < 0.05.

b *p* < 0.001.

Further analyses were conducted to examine the effects of covariates on transitions between different insomnia symptom fluctuation profiles among older adults with cancer pain. In this model, the probability of remaining in the original profile was used as the reference for transition probabilities. The OR represented the relative likelihood of transitioning to a different profile compared with remaining in the same profile. An OR greater than 1 indicated an increased likelihood of transition under the influence of the covariate, whereas an OR less than 1 indicated a decreased likelihood.

As shown in Table 6, increased pain intensity and anxiety levels were significantly associated with a higher likelihood of being classified in the High Insomnia Symptoms profile, compared to the Minimal or No Insomnia Symptoms profile (average pain intensity: OR = 22.29, 95% confidence interval [CI]: 4.04–123.09, *p* < 0.001; anxiety: OR = 1.73, 95% CI: 1.23–2.45, *p* = 0.002), as well as in the Daytime Dysfunction profile compared to the High Insomnia Symptoms profile (average pain intensity: OR = 4.26, 95% CI: 1.22–14.86, *p* = 0.023; anxiety: OR = 3.16, 95% CI: 1.15–8.71, *p* = 0.026).

**Table 6:** Significant covariate effects on short-term transitions in insomnia symptom profiles

Covariates	T1 profile	T2 profile	OR (95% CI)	*p* value
Average pain intensity	Minimal or No Insomnia Symptoms	High Insomnia Symptoms	22.29 (4.04–123.09)	<0.001
Average pain intensity	Daytime Dysfunction	High Insomnia Symptoms	4.26 (1.22–14.86)	0.023
Average pain intensity	High Insomnia Symptoms	Minimal or No Insomnia Symptoms	0.27 (0.10–0.71)	0.008
Average pain intensity	High Insomnia Symptoms	Low Sleep Efficiency	0.28 (0.10–0.78)	0.014
RPFS	Minimal or No Insomnia Symptoms	High Insomnia Symptoms	8.05 (2.04–31.78)	0.003
RPFS	Low Sleep Efficiency	High Insomnia Symptoms	6.50 (1.08–38.94)	0.040
GAD-7	Minimal or No Insomnia Symptoms	High Insomnia Symptoms	1.73 (1.23–2.45)	0.002
GAD-7	Daytime Dysfunction	High Insomnia Symptoms	3.16 (1.15–8.71)	0.026
SPPB	Daytime Dysfunction	Minimal or No Insomnia Symptoms	1.51 (1.13–2.00)	0.005
SPPB	Daytime Dysfunction	Low Sleep Efficiency	1.44 (1.07–1.93)	0.015
SPPB	High Insomnia Symptoms	Minimal or No Insomnia Symptoms	2.15 (1.46–3.16)	<0.001

Only statistically significant associations are shown. OR, odds ratio; CI, confidence interval; RPFS, Revised Piper Fatigue Scale; GAD-7, Generalized Anxiety Disorder 7-item scale; SPPB, Short Physical Performance Battery.

Furthermore, greater fatigue severity was significantly associated with a higher likelihood of being classified in the High Insomnia Symptoms profile at follow-up. Specifically, higher fatigue levels were associated with an increased likelihood of classification from the Minimal or No Insomnia Symptoms profile to the High Insomnia Symptoms profile (RPFS: OR = 8.05, 95% CI: 2.04–31.78, *p* = 0.003), as well as from the Low Sleep Efficiency profile to the High Insomnia Symptoms profile (RPFS: OR = 6.50, 95% CI: 1.08–38.94, *p* = 0.040). In addition, better physical performance (higher SPPB scores) was associated with a higher likelihood of being classified in the Minimal or No Insomnia Symptoms profile (OR = 1.51, 95% CI: 1.13–2.00, *p* = 0.005) or the Low Sleep Efficiency profile (OR = 1.44, 95% CI: 1.07–1.93, *p* = 0.015) at follow-up among individuals initially classified in the Daytime Dysfunction profile.

Finally, improvements in pain and physical performance were significantly associated with more favorable short-term changes in insomnia symptom profiles. Among individuals initially classified in the High Insomnia Symptoms profile, reductions in pain intensity were associated with a higher likelihood of being classified in either the Minimal or No Insomnia Symptoms profile (OR = 0.27, 95% CI: 0.10–0.71, *p* = 0.008) or the Low Sleep Efficiency profile (OR = 0.28, 95% CI: 0.10–0.78, *p* = 0.014) at follow-up. Likewise, better physical performance was significantly associated with a higher likelihood of being classified in the Minimal or No Insomnia Symptoms profile at follow-up (OR = 2.15, 95% CI: 1.46–3.16, *p* < 0.001). The full logistic regression results are provided in Table S1.

## Discussion

The high prevalence of insomnia observed in this study likely reflects the hospitalized, high-risk nature of the sample. Participants were older adults with cancer-related pain assessed during the treatment phase, when hospitalization, pain burden, and psychological stress may worsen sleep. In addition, the AIS is a sensitive screening tool, and the cutoff score of 6 may yield higher prevalence estimates in high-burden clinical populations. Results of the present study identified four subtypes characterizing insomnia symptom fluctuation among older adults with cancer pain (minimal or no insomnia symptoms, daytime dysfunction, low sleep efficiency, and high insomnia symptoms). The LPA and LPTA demonstrated a good fit to these data, suggesting high posterior probabilities within each subtype. The first class, Minimal or No Insomnia Symptoms, was characterized by low scores across all items of the AIS. The second class, Daytime Dysfunction, showed elevated scores on items related to daytime functioning (items 6–8), with lower scores on the remaining items. The third class, Low Sleep Efficiency, exhibited higher scores on nighttime sleep-related items (items 1–5), with relatively lower scores on daytime items. The fourth class, High Insomnia Symptoms, was characterized by high scores across all AIS items. Previous research has supported the multidimensional structure of the AIS, reflecting both nocturnal symptoms (difficulty initiating and maintaining sleep) and daytime dysfunction.^24^^,^^25^ Additionally, studies have shown that distinct components of insomnia symptoms in patients with chronic illnesses (e.g., reduced sleep efficiency and daytime fatigue) reflect different underlying mechanisms of insomnia, characterized by multidimensionality and marked heterogeneity.^26^^,^^27^ The classification identified in the present study highlights the heterogeneity in insomnia symptoms experienced by older adults with cancer pain (see Figures 1 and 2). Patients in the High Insomnia Symptoms profile demonstrated pervasive sleep difficulties affecting both nighttime and daytime functioning. By contrast, those in the Daytime Dysfunction or Low Sleep Efficiency profiles revealed more domain-specific impairments, suggesting that targeted interventions may be necessary to address these distinct patterns of insomnia.

From a clinical perspective, the four insomnia symptom profiles identified in this study suggest that different patient groups may need different treatments. Table 7 shows targeted intervention strategies for each profile. By matching treatments to the specific characteristics of each group, clinicians can create more effective treatment plans.

**Table 7:** Profile-specific management strategies for insomnia

Insomnia symptom profile	Primary characteristics	Primary management strategies
Minimal or No Insomnia Symptoms	few symptoms at night and during the day	- monitor sleep symptoms regularly^5^^,^^28^ - provide basic sleep hygiene education^2^ - maintain regular sleep-wake schedule - encourage light daytime activity
Daytime Dysfunction	significant daytime problems despite less severe nighttime sleep issues	- assess fatigue and emotional distress - encourage structured daytime activity^5^^,^^29^ - optimize pain and symptom control^2^^,^^3^ - provide brief psychological support^3^^,^^29^
Low Sleep Efficiency	significant sleep issues at night (difficulty falling asleep or staying asleep)	- establish consistent sleep schedule^26^^,^^29^ - optimize nighttime pain management^3^ - improve sleep environment conditions^5^ - deliver cognitive behavioral therapy for insomnia (CBT-I)^16^^,^^26^
High Insomnia Symptoms	significant problems with both nighttime sleep and daytime functioning	- deliver cognitive behavioral therapy for insomnia (CBT-I)^5^^,^^29^^,^^30^ - consider short-term pharmacological treatment^31^ - apply relaxation and stress reduction techniques - provide multidisciplinary supportive care^5^ - arrange close follow-up and monitoring

Because the follow-up period was short and only two assessments were available, the findings should be interpreted as reflecting short-term fluctuation in insomnia symptoms and profile classification between the two assessments rather than definitive long-term changes. These shifts likely reflect early responses to hospitalization, initiation of pain treatment, and acute symptom burden. Within this context, the observed insomnia symptom profiles in older adults with cancer pain appeared to show limited short-term variation between the two assessments. Specifically, 73.0% of patients classified as High Insomnia Symptoms were more likely to be classified in the same profile at follow-up, suggesting limited short-term improvement in insomnia symptoms. In contrast, patients in the Minimal or No Insomnia Symptoms, Daytime Dysfunction, and Low Sleep Efficiency profiles showed short-term fluctuation in profile classification, with some individuals being classified in more severe profiles at follow-up. More than 50% of patients initially classified in the Minimal or No Insomnia Symptoms profile were classified in the Daytime Dysfunction profile at follow-up. Additionally, over 40% of individuals in the Daytime Dysfunction profile and more than 30% of those in the Low Sleep Efficiency profile were classified in the High Insomnia Symptoms profile at follow-up. These findings suggest a short-term increase in insomnia symptom burden between the two assessments. These findings are consistent with existing literature, which suggests that insomnia symptoms in cancer patients are dynamic rather than static. Supporting these findings, Kozachik et al.^32^ demonstrated that patterns of pain, fatigue, and insomnia in cancer patients fluctuate over time, and earlier symptom patterns can predict subsequent sleep outcomes. This indicates that insomnia may not remain stable throughout the course of illness or treatment but evolve in response to both physiological and psychosocial factors. Similarly, Al Maqbali et al.^4^ found a high prevalence of insomnia across various stages of cancer, emphasizing that sleep problems are common and may worsen if not continuously monitored and addressed. Daytime Dysfunction and Low Sleep Efficiency may represent clinically important intermediate symptom patterns between Minimal or No Insomnia Symptoms and High Insomnia Symptoms. These stages are characterized by daytime fatigue and reduced nocturnal sleep efficiency. The probabilities of classification into the High Insomnia Symptoms profile at follow-up were 48.1% for individuals initially classified in the Daytime Dysfunction profile and 30.7% for those initially classified in the Low Sleep Efficiency profile. Early identification of individuals with these symptom profiles by clinicians and caregivers may help reduce the likelihood of short-term worsening in insomnia symptoms. In addition, the proportion of individuals classified in the High Insomnia Symptoms profile was slightly higher at follow-up, and more than 70% of those initially classified in this profile were classified in the same profile at the second assessment, despite not receiving sedative or hypnotic medication. This suggests that older patients with cancer classified in the High Insomnia Symptoms profile had limited short-term improvement in insomnia symptom burden between the two assessments. These findings are consistent with those of Lowery-Allison et al.,^33^ who reported that insomnia initiated or worsened during cancer treatment persisted for up to 10 years in breast cancer survivors after treatment cessation. Similarly, a review by Al Maqbali^4^ emphasized that conventional sleep hygiene practices or general interventions are often insufficient for patients with severe insomnia. In line with these results, Yang et al.^34^ demonstrated that predictors of insomnia change over time, with anxiety being a key factor in the early treatment phase, while fatigue and perceived stress became more prominent in the later stages. Collectively, these studies suggest that insomnia is dynamic and evolve in response to both physiological and psychological factors. They also highlight the importance of longitudinal assessment, as single-time-point evaluations may underestimate the risk of worsening sleep problems. The present study extends these findings by estimating profile classification probabilities between two assessments in older patients with cancer, providing a more nuanced understanding of short-term fluctuation in insomnia symptoms and helping identify individuals at higher risk. Clinically, these results underscore the necessity of proactive, subtype-specific interventions to manage short-term changes in insomnia and improve quality of life in this vulnerable population.

The study shows that insomnia reduce the quality of life in older adults with cancer pain; therefore, prevention and treatment of insomnia are crucial for this population. To better understand this issue, it is important to explore the factors influencing the short-term changes in insomnia symptom burden among elderly cancer patients. Our findings indicate that higher baseline pain, fatigue, and anxiety were linked to a higher chance of moving into the High Insomnia Symptoms profile over the short follow-up period. In contrast, better baseline physical function was linked to a lower chance of this change. All of the covariates examined earlier were measured at baseline and used as predictors of short-term changes in insomnia symptoms. Several biological and behavioral mechanisms may help explain the association between baseline pain intensity and short-term worsening of insomnia. These include increased nighttime pain, heightened physiological arousal, and inflammatory activity (e.g., interleukin-6 [IL-6], tumor necrosis factor alpha [TNF-α]).^5^ In addition, sleep deprivation lowers the pain threshold, which may further exacerbate insomnia symptom burden. Recent clinical evidence and guideline recommendations consistently highlight the strong association between insomnia and pain in cancer patients, which becomes more pronounced in the context of cancer pain management, particularly with opioid use.^35^^,^^36^ Our results are in line with the 2023 ESMO Guidelines on Cancer-Related Insomnia,^29^ as well as findings from recent real-world and retrospective studies^5^^,^^36^

Moreover, patients with higher baseline levels of CRF were more likely to fluctuate to the High Insomnia Symptoms profile during the short follow-up period. This finding is in line with earlier studies that reported similar symptom changes. Epidemiological evidence indicates high rates of comorbidity between fatigue and insomnia in oncology populations, with both conditions often mutually reinforcing each other and impairing quality of life.^37^ Fatigue exacerbates insomnia through multiple mechanisms, including disrupting circadian rhythms, persistently activating inflammatory pathways, and heightening pre-sleep arousal. These processes lead to delayed sleep onset and increased nocturnal awakenings.^16^ Conversely, insomnia increases energy expenditure and impairs immune function. This amplification of fatigue establishes a self-perpetuating cycle.^38^ Previous studies have shown that baseline fatigue is associated with later declines in sleep quality and a higher risk of insomnia, which is consistent with the associations observed in the present study.^17^ These results suggest that fatigue not only represents a common concomitant symptom but also serves as an important predictor of worsening insomnia in older adults with cancer pain. Consequently, clinical management should consider fatigue and insomnia as an interrelated symptom cluster; this warrants integrated intervention strategies.

The average GAD-7 score in this sample showed a moderate level of anxiety. This level is often seen in older adults who have cancer pain. It likely comes from being in the hospital, dealing with ongoing pain, and feeling stress related to cancer. Other studies have found similar anxiety levels in people with cancer who are receiving care.^39^ This study found that higher levels of anxiety were associated with a greater likelihood of being classified in the High Insomnia Symptoms profile at follow-up. Anxiety may increase physical arousal before sleep, leading to delayed sleep onset and more frequent nocturnal awakenings.^29^^,^^36^ Furthermore, anxious tendencies may cause patients to interpret pain as more threatening, thereby elevating pain catastrophizing. This pain catastrophizing amplifies pain perception and anticipatory distress.^40^ As a result, it further disrupts sleep through emotional arousal and stress responses. Empirical evidence has consistently demonstrated that anxiety not only coexists with insomnia but also that anxiety-related cognitive biases can partially mediate or predict the persistence and exacerbation of insomnia.^41^ Moreover, pain catastrophizing in cancer populations has been shown to be strongly associated with pain intensity, emotional distress, and impaired sleep quality. It functions as both a mediator and moderator, thereby exacerbating the relationship between chronic pain and insomnia.^42^ Together, these findings underscore the importance of targeting both anxiety and pain catastrophizing in the prevention and management of insomnia among older adults with cancer pain, with the goal of breaking the negative feedback loop between anxiety and insomnia.

This study demonstrated that better physical function was associated with a reduced risk of short-term worsening of insomnia symptom burden. Higher levels of physical function generally reflect greater daytime activity,^43^ more stable circadian rhythms,^44^ and lower systemic inflammation.^45^ These factors enhance homeostatic sleep pressure and nocturnal sleepiness, thereby mitigating the risk of worsening insomnia. In a prospective cohort study of stage I–III colorectal cancer survivors, superior physical performance and activity rhythms were linked to fewer insomnia symptoms, improved functional status, and higher quality of life.^20^ Similarly, Sagarra-Romero reported that exercise interventions, whether home based or outpatient, improved sleep quality by reducing sleep latency and increasing total sleep time and sleep efficiency.^46^ Collectively, these findings suggest that maintaining physical function and staying physically active can reduce cancer-related pain and related symptoms. These behaviors may also help limit short-term worsening of insomnia by supporting normal sleep balance and daily sleep rhythms.^47^ Consistent with these results, a meta-analysis by Gururaj et al.^43^ indicated that better physical fitness significantly improved sleep quality, sleep latency, and circadian rhythms in cancer survivors. However, Gururaj also noted that the effects of exercise on sleep efficiency, total sleep duration, sleep latency, and circadian rhythm parameters were not statistically significant, potentially due to variations in intervention type, intensity, and duration.^43^ These studies collectively underscore the importance of preserving and enhancing physical function in older adults with cancer pain. From a clinical perspective, these findings suggest that maintaining physical function may be relevant for reducing the risk of short-term worsening of insomnia. As tolerated, older adults with cancer pain may benefit from moderate physical activity and individualized rehabilitation programs.

### Limitations of the study

This study has several limitations that should be considered when interpreting the findings. First, the two-center regional design and relatively small sample size may limit generalizability. Second, although latent transition analysis captures short-term changes, the 7–10 day follow-up does not permit inference about long-term insomnia trajectories. The findings should therefore be interpreted as short-term symptom dynamics. Future longitudinal studies with larger, more diverse cohorts are warranted to better elucidate the directionality and causality of associations between insomnia, pain intensity, fatigue, anxiety, and physical functioning. Third, although we calculated the weekly cumulative morphine-equivalent dose to account for the analgesic burden, we did not systematically track the specific types of individual analgesics beyond this cumulative measure. Different analgesic regimens may influence sleep quality in distinct ways, and future studies should consider systematically recording analgesic types to better understand their potential impact on sleep outcomes.

Fourth, the high rate of insomnia in this study should be understood in light of the study population. The participants were older adults with cancer-related pain, and factors such as adverse symptoms and the hospital environment may disrupt sleep. In addition, the AIS is also a sensitive screening measure. Its commonly used cutoff score of 6 may lead to higher estimates of insomnia symptoms in clinical samples with heavy symptom burden. Moreover, the AIS assesses symptoms over the past month, whereas the interval between T1 and T2 was at most 2 weeks. This mismatch may have introduced overlap in recall periods and limited the independence of the two assessments, potentially affecting the estimation of short-term changes. Future studies may benefit from using instruments with shorter recall periods, such as the Insomnia Severity Index (ISI) or daily sleep diaries, to better capture short-term fluctuations. The study population was from Fujian Province. Local factors, such as access to healthcare, regional healthcare practices, and cultural attitudes toward illness and treatment, may affect how these findings apply to other regions or countries. These factors can shape the healthcare experience for cancer patients and influence the prevalence and management of insomnia. Fifth, depression is common in patients with cancer and may overlap with symptoms of insomnia and anxiety. Future studies should include standardized measures of depression to better clarify its role in insomnia changes. This study also did not use objective tests to diagnose insomnia. It also left out several factors that may matter. These factors include cancer treatments, comorbid conditions, medication use, social support, and coping behaviors. Future studies should add these objective tests and these key factors. This approach can help explain what drives insomnia and how it changes in this group.

Despite these limitations, this study also has notable strengths. To our knowledge, this study is among the few employing latent transition analysis to characterize dynamic changes in insomnia symptom burden among older adults with cancer pain. Moreover, the statistical approach allowed for the identification of transition probabilities between distinct insomnia profiles, providing a more detailed understanding of short-term changes in insomnia symptoms.

Clinically, the results highlight the importance of early assessment and continuous monitoring of insomnia in older cancer patients. Interventions targeting modifiable determinants such as pain management, fatigue mitigation, anxiety reduction, and enhancement of physical functioning may help reduce the risk of short-term worsening of insomnia.^46^^,^^48^ Strategies could include individualized pain control plans, graded physical activity programs, cognitive-behavioral therapy for insomnia, relaxation training, and structured psychosocial support. Healthcare providers should incorporate routine sleep assessments into clinical practice and consider multidisciplinary collaboration, including oncology, nursing, rehabilitation, and psychosocial services.^28^ These holistic approaches may improve sleep quality, overall well-being, and resilience in older patients with cancer pain. Further studies are needed to establish their definitive clinical impact.

This study identified four distinct insomnia profiles among older adults with cancer pain using LPA: minimal or no insomnia symptoms, daytime dysfunction, low sleep efficiency, and high insomnia symptoms. LPTA also further revealed the dynamic nature of these profiles and highlighted key factors linked to short-term changes between profiles. Higher pain intensity, fatigue, and anxiety were associated with an increased risk of short-term worsening of insomnia symptom burden. In contrast, better physical functioning was protective against short-term worsening of insomnia symptom burden. These findings indicate that insomnia in this population is not static but evolves with changes in symptom burden and functional status, underscoring the importance of continuous monitoring. Based on these findings, comprehensive symptom management—particularly targeting pain, fatigue, and anxiety—may help prevent the risk of short-term worsening of insomnia. Additionally, strategies to maintain or enhance physical functioning are important. Multidisciplinary collaboration, integrating oncology, nursing, rehabilitation, and psychosocial care, is recommended to optimize sleep health and overall quality of life.

This study provides insights into short-term fluctuation in insomnia symptom profiles among older adults with cancer pain; however, it is limited by the lack of treatment-related data and objective diagnostic measures. Future research should explore broader determinants, such as treatment side effects, coping strategies, and social support, and assess targeted interventions for insomnia symptoms as well as physical activity programs. Proactive, holistic approaches may help preserve sleep quality, enhance resilience, and improve overall well-being in this vulnerable population.

## Resource availability

### Lead contact

Further information regarding this manuscript and requests should be directed to the lead contact, Yunzhen Peng (fjpyz114@163.com).

### Materials availability

This study did not generate new unique reagents.

### Data and code availability

• De-identified participant-level data have been deposited in Zenodo and are available at https://doi.org/10.5281/zenodo.20350706. Access is restricted because the dataset contains potentially sensitive clinical information. Access requests may be submitted through Zenodo and will be reviewed subject to applicable institutional and ethical requirements.
• All custom analysis code used in this study has been deposited in Zenodo and is available at https://doi.org/10.5281/zenodo.18607337.
• Any additional information required to reanalyze the data reported in this paper is available from the lead contact upon request.

## Acknowledgments

The authors would like to sincerely thank all the patients and researchers who participated in this study. The authors gratefully acknowledge the Fujian Provincial Traditional Chinese Medicine Science and Technology Program Foundation (no. 2025YBB016), the University-Administered Project of Fujian University of Traditional Chinese Medicine (no. XB2024191), Natural Science Foundation of Fujian Province of China (no. 2023J011241), Natural Science Foundation of Fujian Province of China (no. 2024J011104), and the Joint Funds for the Innovation of Science and Technology, Fujian Province (no. 2025Y9669).

## Author contributions

Conceptualization, Y.P.; methodology, Y.P., H.Z., and R.Q.; software, H.Z. and R.Q.; investigation, H.Z., H.L., H.C., M.L., W.H., and L.J.; data curation, H.Z. and R.Q.; writing – original draft, H.Z. and R.Q.; writing – review and editing, H.Z., R.Q., H.L., X.K., and Y.P.; visualization, H.Z. and R.Q.; supervision, X.K. and Y.P.; project administration, Y.P.; funding acquisition, Y.P. and X.K.

## Declaration of interests

The authors declare no competing interests.

## Declaration of generative AI and AI-assisted technologies in the writing process

During the preparation of this work, the authors used DeepSeek for language editing and improving clarity of expression. The authors reviewed and edited the content thoroughly and take full responsibility for the content of the publication.

## STAR★Methods

### Key resources table

**Table:** 

REAGENT or RESOURCE	SOURCE	IDENTIFIER
**Deposited data**		

De-identified participant dataset	This paper	Zenodo: https://doi.org/10.5281/zenodo.20350706

**Software and algorithms**		

Custom analysis code	Zenodo	https://doi.org/10.5281/zenodo.18607337
Mplus version 7.4	Muthén & Muthén	https://www.statmodel.com
SPSS version 23.0	IBM	https://www.ibm.com/products/spss-statistics

### Experimental model and study participant details

#### Participants and study design

A prospective observational cohort study was conducted among older adults with cancer-related pain in two tertiary cancer centers in Fujian Province, China, between April 2023 and April 2025. These centers provide integrated oncology, pain management, and supportive care services with a multidisciplinary approach to symptom management.

Eligible participants were aged 60 years or older, clinically diagnosed with malignancy and experiencing cancer-related pain (defined as an average pain score ≥1 on the Numeric Rating Scale [NRS]). Additional inclusion criteria required patients to have a stable condition, with an expected ability to complete two follow-up assessments, and to provide written informed consent prior to enrollment. Exclusion criteria included: presence of psychiatric disorders, agitation, or dementia; cognitive or language impairments preventing effective communication; current use of sedative-hypnotic medications; or neurological conditions known to affect sleep or cognition, such as Parkinson’s disease, stroke, epilepsy, or other neurodegenerative disorders. Additionally, patients with brain metastases or primary central nervous system tumors were also excluded, as their sleep disturbances may arise through distinct mechanisms.

All patients received standard pharmacologic pain management according to institutional protocols and national guidelines for cancer pain treatment. Treatment was initiated as part of routine care, and the timing of initiation varied depending on each patient’s clinical status and needs.

#### Data collection and sample characteristics

Data were collected at two time points: within 48 h of hospital admission (T1) and 7–10 days after initiation of in-hospital pain management or routine supportive treatment (T2). Prior to enrollment, trained investigators explained the study procedures and obtained written informed consent from all participants. Most participants had at least a primary or secondary level of education. When necessary, assistance was provided by family members or research staff to facilitate communication and ensure completion of the questionnaires. A total of 280 patients were enrolled at T1. The team excluded 27 patients who were using sleep medications. Two patients withdrew from the study, and three were transferred to other institutions. In the end, 238 participants completed assessments at both time points. The mean age of participants was 67.94 years (SD = 6.12; range 60–95). Most participants were male (60.5%). More than half (59.2%) lived with family members.

#### Ethics statement

The study protocol was approved by the Ethics Committee of Fujian Cancer Hospital (Approval No. K2023-271-01) and was conducted in accordance with the Declaration of Helsinki and relevant regulations. Written informed consent was obtained from all participants before enrollment. All participants were aged 60 years or older and were of Han Chinese ethnicity. Age, sex, and ethnicity were collected at baseline; age and sex were summarized descriptively.Sex was examined as a covariate in the latent profile transition model.

### Method details

#### Instruments

*Numeric Rating Scale (NRS).* Pain intensity was measured using NRS, an 11-point scale ranging from 0 (no pain) to 10 (worst imaginable pain), which has demonstrated strong reliability and validity in cancer populations, including older adults.^49^ The participants were asked to select the number that best represented their pain intensity, and the number selected represented their NRS score. As a single-item instrument, internal consistency reliability is not applicable. The NRS has demonstrated good test–retest reliability, validity, and responsiveness in cancer populations.

*The Athens Insomnia Scale (AIS).* was used as a screening instrument for clinical insomnia symptoms rather than as a diagnostic tool for insomnia disorder. The AIS is a self-reported instrument developed to assess sleep difficulties in accordance with the ICD-10 criteria.^24^ It comprises eight items, each rated on a 0(no problem at all) to 3 (a very serious problem), yielding a total score ranging from 0 to 24. According to the recommended cutoff, AIS scores >6 were interpreted as indicating clinically relevant insomnia symptoms. This cutoff is commonly used for insomnia screening and provides a balance between sensitivity (93%) and specificity (85%).^24^^,^^50^ The Chinese version of the AIS has demonstrated strong reliability and validity in both research and clinical contexts.^51^ In the current sample, the Chinese version of the AIS demonstrated good internal consistency, with a Cronbach’s alpha of 0.702 at baseline and 0.706 at follow-up. AIS assesses symptoms over the past month, whereas the interval between T1 and T2 was at most two weeks, creating some overlap in recall periods, the AIS was chosen for its strong psychometric properties and high reliability. The AIS provides a comprehensive assessment of both nocturnal and daytime symptoms, which are crucial for understanding the overall impact on patients’ quality of life.

*The Revised Piper Fatigue Scale (RPFS)*. The Piper Fatigue Scale (PFS), originally developed in 1987^52^ and revised in 1998,^53^ consists of 22 items across four dimensions: sensory (5 items), affective (5 items), cognitive (6 items), and behavioral (6 items). Each item is rated on an 11-point scale from 0 (“no change”) to 10 (“severe change”). The total fatigue score is calculated as the mean of the four dimension scores and categorized as mild (0–3.3), moderate (3.4–6.7), or severe fatigue (6.8–10). The scale is widely used due to its simplicity and applicability.^53^ The Chinese version has demonstrated excellent psychometric properties, with a test-retest reliability of 0.98, Cronbach’s alpha of 0.91, and validity coefficient of 0.92.^54^ In the current sample, the Cronbach’s alpha was 0.766.

*Generalized Anxiety Disorder 7-item scale (GAD-7).* GAD-7 was developed by Spitzer et al.^39^ based on the Diagnostic and Statistical Manual of Mental Disorders, Fourth Edition (DSM-IV). It is a brief self-report instrument designed to assess anxiety symptoms. The scale consists of seven items, each rated on a 4-point Likert scale (0 = not at all, 1 = several days, 2 = more than half the days, 3 = nearly every day), with total scores ranging from 0 to 21. Scores ≥5 indicate possible anxiety, with severity categorized as mild (5–9), moderate (10–14), and severe (15–21). The GAD-7 demonstrates high reliability and validity for anxiety screening, with a reported Cronbach’s alpha of 0.90.^39^^,^^55^ In the current sample, the Cronbach’s alpha was 0.743.

*The Short Physical Performance Battery (SPPB)*. The SPPB developed by the National Institute on Aging,^56^ has demonstrated high validity and sensitivity in assessing physical function among older community-dwelling adults.^57^ The SPPB comprises three tests: gait speed measured over a 2.5-meter walk, balance assessment, and five repeated chair stands. Each component is scored from 0 (worst performance) to 4 (best performance), yielding a total score ranging from 0 to 12. Higher scores indicate better physical function, with scores categorized as poor (0–6), moderate (7–9), and good (10–12). The scale’s internal consistency reliability has been reported with a Cronbach’s alpha of 0.89.^56^^,^^57^ In the current sample, the Cronbach’s alpha was 0.784.

#### Covariates

Beyond the factors previously described, the effects of sex, living arrangements, average pain intensity, and weekly cumulative morphine-equivalent dose on insomnia among elderly cancer pain patients were investigated. Covariates were selected *a priori* based on existing literature and clinical considerations. Pain intensity,^5^ cancer-related fatigue,^16^ anxiety,^18^ physical functioning,^20^ and opioid use^14^ have been consistently linked to insomnia in cancer pain populations. In addition, sex and living arrangement were included because demographic characteristics and social context may influence sleep and psychosocial functioning in older adults.^13^ All covariates were assessed at baseline (T1) and were treated as affect baseline predictors of insomnia symptom profiles.

### Quantification and statistical analysis

Descriptive statistics were used to summarize participant characteristics and symptom severity. Continuous variables are presented as mean and standard deviation (SD), and categorical variables are presented as frequencies and percentages. Differences in the prevalence of positive responses on individual Athens Insomnia Scale (AIS) items and AIS total scores >6 between T1 and T2 were examined using chi-square tests, with χ^2^ values and *p* values reported.

Latent Profile Analysis (LPA) was used to identify insomnia symptom profiles at each assessment point. Model selection was based on the Akaike Information Criterion (AIC), Bayesian Information Criterion (BIC), adjusted BIC (aBIC), entropy, Lo-Mendell-Rubin likelihood ratio test (LMR), and Bootstrap Likelihood Ratio Test (BLRT). Lower information criteria values and higher entropy values indicated better model fit, while theoretical interpretability and model parsimony were also considered.

Latent Profile Transition Analysis (LPTA), an extension of LPA for longitudinal data, was used to examine short-term changes in insomnia symptom profile membership across the two assessments. Transition probabilities were estimated to describe the likelihood of remaining in the same profile or moving to another profile from T1 to T2.

To examine factors associated with insomnia symptom profiles and short-term profile transitions, multinomial logistic regression analyses were performed. Covariates were selected *a priori* based on previous literature and clinical relevance and included sex, living arrangements, average pain intensity, weekly cumulative morphine-equivalent dose, fatigue measured by the Revised Piper Fatigue Scale (RPFS), anxiety measured by the Generalized Anxiety Disorder 7-item scale (GAD-7), and physical performance measured by the Short Physical Performance Battery (SPPB). Odds ratios (ORs), 95% confidence intervals (CIs), exact *p* values, and the number of participants were reported where applicable. In Figures 1 and 2, points represent class-specific estimated mean AIS item scores, and error bars represent standard errors (SEs).Statistical significance was set at a two-sided *p* < 0.05. No asterisk-based significance annotations were used in the figures.

All latent profile and transition analyses were performed using Mplus version 7.4, while descriptive statistics and multinomial logistic regression analyses were conducted using SPSS version 23.0 (IBM SPSS Statistics, Armonk, NY, USA).

## Supplemental information

Table S1. Full logistic regression coefficients and odds ratios for the LPTA model with covariates of average pain intensity, fatigue, anxiety, and physical functioning, related to Table 6
