# Supplementary material for: Short-term insomnia symptom profile transitions in older patients with cancer pain
Source: iScience. 2026 Jul 1;29(7):116557. doi: 10.1016/j.isci.2026.116557 (PMC13378372; doi:10.1016/j.isci.2026.116557)
Supplement: Table S1. Full logistic regression coefficients and odds ratios for the LPTA model with covariates of average pain intensity, fatigue, anxiety, and physical functioning, related to Table 6 [file mmc1.pdf]

iScience, Volume *29*

Supplemental information

Short-term insomnia symptom profile transitions in older patients with cancer pain

Hongyu Zhu, Rong Qi, Huiyu Luo, Huiqun Chen, Meiqun Lin, Xi Ke, Wenting Huang, Liqin Jiang, and Yunzhen Peng

Supplementary Table S1. Full logistic regression coefficients and odds ratios for the LPTA model with covariates of average pain intensity, fatigue, anxiety, and physical functioning

**Covariates**	**Latent status**	**Minimal or No Insomnia Symptoms**		**Daytime Dysfunction**		**Low Sleep Efficiency**		**High Insomnia Symptoms**	
		OR (95% CI)	p-value	OR (95% CI)	p-value	OR (95% CI)	p-value	OR (95% CI)	p-value
Average pain intensity	Minimal or No Insomnia Symptoms	REF	REF	0.91 (0.46, 1.79)	0.787	1.60 (0.18, 14.47)	0.676	0.27 (0.10, 0.71)	0.008
Average pain intensity	Daytime Dysfunction	3.16 (0.73, 13.66)	0.123	REF	REF	4.62 (0.33, 65.13)	0.257	0.48 (0.18, 1.26)	0.136
Average pain intensity	Low Sleep Efficiency	3.78 (0.90, 15.88)	0.069	1.07 (0.54, 2.12)	0.856	REF	REF	0.28 (0.10, 0.78)	0.014
Average pain intensity	High Insomnia Symptoms	22.29 (4.04, 123.09)	<0.001	4.26 (1.22, 14.86)	0.023	7.75 (0.69, 86.45)	0.096	REF	REF
RPFS	Minimal or No Insomnia Symptoms	REF	REF	0.73 (0.51, 1.04)	0.080	1.44 (0.49, 4.28)	0.511	1.11 (0.70, 1.77)	0.660
RPFS	Daytime Dysfunction	3.06 (0.82, 11.48)	0.097	REF	REF	1.59 (0.43, 5.95)	0.490	0.77 (0.48, 1.23)	0.275
RPFS	Low Sleep Efficiency	3.58 (0.99, 12.94)	0.051	0.96 (0.67, 1.37)	0.808	REF	REF	1.31 (0.75, 2.28)	0.349
RPFS	High Insomnia Symptoms	8.05 (2.04, 31.78)	0.003	4.03 (0.96, 16.96)	0.057	6.50 (1.08, 38.94)	0.040	REF	REF
GAD-7	Minimal or No Insomnia Symptoms	REF	REF	0.94 (0.80, 1.11)	0.478	1.23 (0.75, 2.03)	0.406	0.94 (0.76, 1.17)	0.570
GAD-7	Daytime Dysfunction	1.08 (0.82, 1.42)	0.583	REF	REF	1.06 (0.59, 1.90)	0.843	1.12 (0.88, 1.43)	0.361
GAD-7	Low Sleep Efficiency	1.26 (0.97, 1.64)	0.080	1.06 (0.89, 1.26)	0.530	REF	REF	0.99 (0.79, 1.27)	0.985
GAD-7	High Insomnia Symptoms	1.73 (1.23, 2.45)	0.002	3.16 (1.15, 8.71)	0.026	1.68 (0.91, 3.07)	0.095	REF	REF
SPPB	Minimal or No Insomnia Symptoms	REF	REF	1.51 (1.13, 2.00)	0.005	1.91 (0.81, 4.48)	0.138	2.15 (1.46, 3.16)	<0.001
SPPB	Daytime Dysfunction	1.19 (0.76, 1.86)	0.440	REF	REF	0.98 (0.36, 2.67)	0.981	1.11 (0.75, 1.66)	0.601
SPPB	Low Sleep Efficiency	1.20 (0.80, 1.79)	0.377	1.44 (1.07, 1.93)	0.015	REF	REF	1.35 (0.91, 1.99)	0.141
SPPB	High Insomnia Symptoms	1.28 (0.65, 2.52)	0.481	0.49 (0.21, 1.16)	0.103	0.56 (0.22, 1.43)	0.222	REF	REF
